# CAFOs and Environmental Justice: The Case of North Carolina

**DOI:** 10.1289/ehp.121-a182

**Published:** 2013-06-01

**Authors:** Wendee Nicole

**Affiliations:** Wendee Nicole, based in Houston, TX, has written for *Nature*, *Scientific American*, *National Wildlife*, and other magazines.

On the coastal plain of eastern North Carolina, families in certain rural communities daily must deal with the piercing, acrid odor of hog manure—reminiscent of rotten eggs and ammonia—wafting from nearby industrial hog farms. On bad days, the odor invades homes, and people are often forced to cover their mouths and noses when stepping outside. Sometimes, residents say, a fine mist of manure sprinkles nearby homes, cars, and even laundry left on the line to dry.^1^

Today’s industrial-scale farms—called concentrated animal feeding operations (CAFOs)—house thousands of animals whose waste is periodically applied to “spray fields” of Bermuda grass or feed crops.^2^^,^^3^ The waste can contain pathogens, heavy metals, and antibiotic-resistant bacteria,^4^^,^^5^ and the spray can reach nearby homes and drinking water sources. The odor plume, which often pervades nearby communities, contains respiratory and eye irritants including hydrogen sulfide and ammonia.^6^^,^^7^^,^^8^ A growing body of research suggests these emissions may contribute not only to mucosal irritation^9^ and respiratory ailments^10^ in nearby residents but also decreased quality of life,^11^ mental stress,^12^^,^^13^ and elevated blood pressure.^14^

**Figure:**
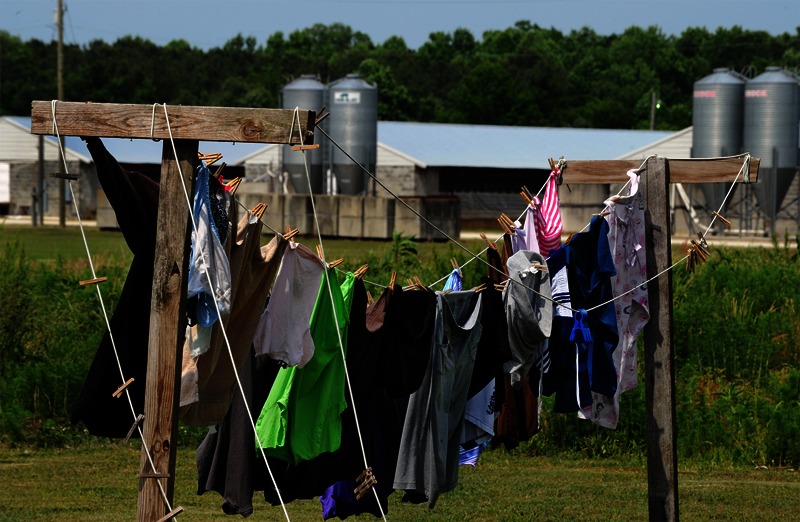
The home of a minority family in Kenansville, North Carolina, situated next to a concentrated animal feeding operation, or CAFO. Dust, odors, and manure from CAFOs can reach nearby residents’ homes, including their laundry. ©2013 Donn Young Photography

Although the Midwest is the traditional home for hogs, with Iowa still the top-producing state, North Carolina went from fifteenth to second in hog production between the mid-1980s and mid-1990s.^15^ This explosive growth resulted in thousands of CAFOs located in the eastern half of the state—squarely in the so-called Black Belt, a crescent-shaped band throughout the South where slaves worked on plantations.^16^^,^^17^ After emancipation many freed slaves continued to work as sharecroppers and tenant farmers. A century later, black residents of this region still experience high rates of poverty, poor health care, low educational attainment, unemployment, and substandard housing.^18^^,^^19^

The clustering of North Carolina’s hog CAFOs in low-income, minority communities—and the health impacts that accompany them—has raised concerns of environmental injustice and environmental racism.^20^ As one pair of investigators explained, “[P]eople of color and the poor living in rural communities lacking the political capacity to resist are said to shoulder the adverse socio-economic, environmental, or health related effects of swine waste externalities without sharing in the economic benefits brought by industrialized pork production.”^21^ Although North Carolina is not the only area with environmental justice concerns vis-à-vis CAFOs, it has become one of the best studied.

## Environmental Injustice?

One of the misunderstandings about environmental racism, in particular, is that the term suggests malicious or at least discriminatory intent in terms of locating hazards. Although that may exist in some cases, several studies have argued that industry or government simply followed the “path of least resistance” in choosing sites where people were less likely to object or land was cheap.^22^^,^^23^ The situation nevertheless results in environmental injustice if minority populations are disproportionately affected, no matter the reason.^24^

From a scientific perspective, hundreds of studies have documented disparities in the location of environmental hazards relative to race and class, and, further, in the extent and timeliness of remediative actions.^25^^,^^26^^,^^27^^,^^28^ “Environmental justice science [seeks to] understand how burden disparities lead to exposure, risk, and health disparities,” says Sacoby Wilson, a University of Maryland environmental health professor.

**Figure:**
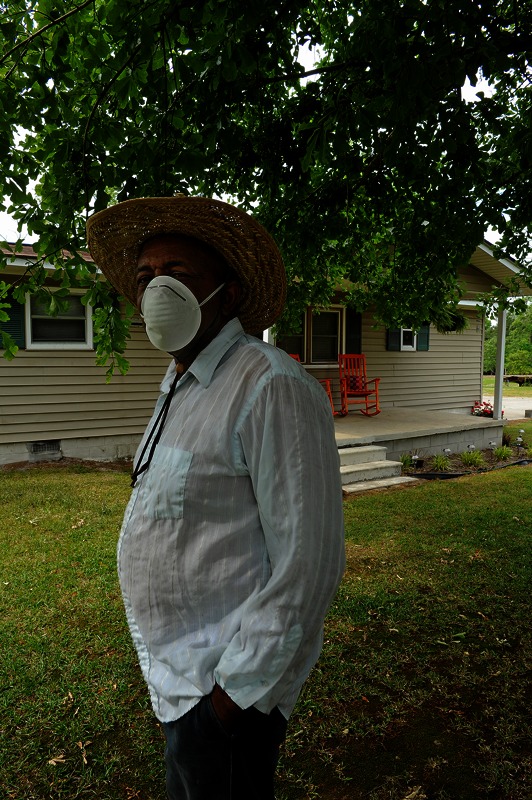
This resident of Kenansville usually wears a facemask when he’s in his yard because of the dust from the neighboring CAFO. Most studies suggest that communities already have high levels of poverty and large percentages of minority residents when CAFOs are built there. People who can afford to move away often do. ©2013 Donn Young Photography

Debates still exist over the relative importance of race versus socioeconomic status^29^ and whether hazards are disproportionately sited in regions where minorities and impoverished people live, or whether communities change after polluting facilities move in. Most studies suggest the former.^22^^,^^30^ However, research also suggests that people who can afford to move away from environmental hazards often do, increasing disparities.^30^

East Carolina University sociology professor Bob Edwards says he had heard environmental justice groups claiming disparities in the siting of hog farms and industry proponents denying them when he realized it was an empirical question. “There was no real research at the time,” he says. So in 2000 he began a study with collaborator Anthony E. Ladd of the Loyola University Department of Sociology. They found that even when controlling for regional differences, urbanization level, property value, and attributes of the labor force, eastern North Carolina counties with larger minority populations were home to greater concentrations of hog waste, a function of hog population density, compared with more urbanized counties with a higher percentage of white residents.^21^ Another North Carolina study later reported nine times more hog CAFOs in areas where there was more poverty and higher percentages of nonwhite people even after adjusting for population density as a measure of rural location and cheaper land.^20^

**Figure:**
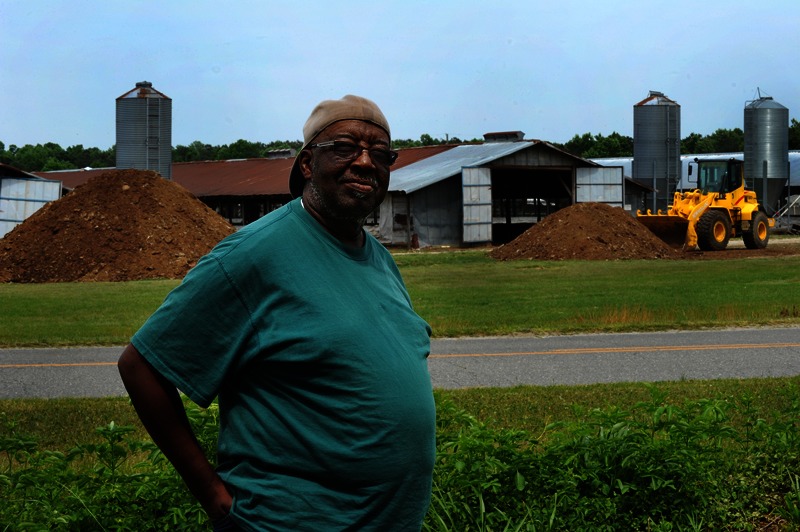
Another Kenansville resident stands in his front yard, feet from the CAFO across the street. Donn Young, the North Carolina–based photographer who took these images, says of his time in Kenansville, “I encountered problems with my eyes—itchy, watering, something akin to allergies.” To the people who live there, CAFOs are simply a fact of everyday life. ©2013 Donn Young Photography

Edwards has also reported that large hog operations forced small farmers out of business.^31^ As the industry consolidated, the primary slaughterhouse in North Carolina refused to accept hogs in lots of fewer than 1,000.^32^ With the exception of the slaughterhouse, the industry does not create many working-class jobs and sometimes creates major rifts in the social fabric of communities between proponents and opponents of local CAFOs.^31^^,^^33^^,^^34^^,^^35^

**What Is Environmental Justice?**

Environmental justice refers to both a social movement and a field of scientific research. As a movement, it is a marriage of civil rights and environmentalism, emerging in 1982 when black citizens lay down on the road to stop the government from dumping 120 million pounds of soil contaminated with polychlorinated biphenyls in Warren County, North Carolina.74 Although the Warren County waste site ultimately was established,^75^^,^^76^ the protests captured the nation’s attention.

The study of environmental justice began in earnest in 1983, when the Government Accountability Office (then known as the General Accounting Office) investigated the racial composition of communities near four hazardous waste sites in the Southeast, three of which were located in predominantly black communities where at least 26% of the population lived below the poverty level.^77^ In 1987 the first national study to analyze the issue with multivariate statistics found that even after controlling for household income, housing values, amount of hazardous waste generated in an area, and other factors, the percentage of minority residents in a zip code proved the greatest predictor of hazardous waste facility siting. Zip codes with hazardous waste sites had double the percentage of minority residents compared with those with none, and zip codes with more than one facility had triple the percentage of minority residents.^74^

By the early 1990s, the federal government first acknowledged environmental justice with a working group that published the report Environmental Equity: Reducing Risks for All Communities.^78^ Soon after, the Environmental Protection Agency created the Office of Environmental Equity, since renamed the Office of Environmental Justice. The agency defines environmental justice as “the fair treatment and meaningful involvement of all people regardless of race, color, national origin, or income with respect to the development, implementation, and enforcement of environmental laws, regulations, and policies.”^79^

## A Brief History of Swine

For centuries, animal husbandry operated much like a farm in a cartoon: pigs wallowing in mud, chickens wandering about pecking the ground, and cows grazing on grass, with a barn to store hay and feed. Farms were largely sustainable; they generally did not deplete the soil, water, or land resources needed to maintain the farm for the next generation. The waste from the animals helped grow the next year’s crops.

Today, the vast majority of America’s 1 billion–plus food animals slaughtered annually^36^ are raised in CAFOs.^37^ John Ikerd, professor emeritus of agricultural economics at the University of Missouri, says farms have changed over his long career in three main ways. First, today’s farms specialize in growing one crop or in one phase of production; in the hog industry there are facilities for breeding sows, raising piglets to about 40 pounds, and finishing operations, where animals are raised to the point of slaughter. Second, large corporations (“integrators”) contract with individual farmers to raise animals and set precise standards for what the animals eat, their housing conditions, and the antibiotics and hormones they receive. Finally, there’s been a consolidation of control and ownership that, as mentioned, has forced small farmers out of business and altered local economies and communities.^31^^,^^32^

The hog industry in North Carolina changed rapidly starting in the 1970s, when Wendell Murphy applied the CAFO model, already used for poultry, to swine.^38^ As a successful hog farmer, Murphy was elected to the North Carolina House of Representatives in 1983 and to the state Senate in 1988, where he sponsored and helped to pass legislation—dubbed “Murphy’s laws”—that eliminated sales tax on hog farm equipment and prevented local authorities from using zoning authority to deal with odor issues.^39^^,^^40^

**Figure:**
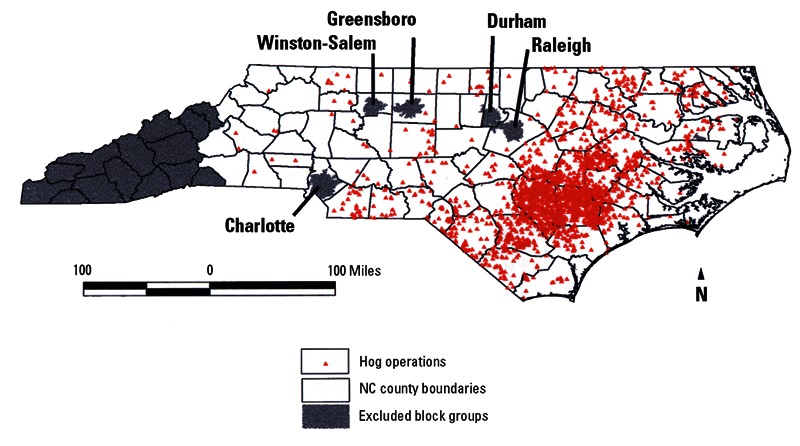
Maps from an older study show distributions of poverty, minority residents, and hog CAFOs in North Carolina as of 1998–2000. Little has changed appreciably since then. Source: Wing et al. (2000)^20^

**Figure:**
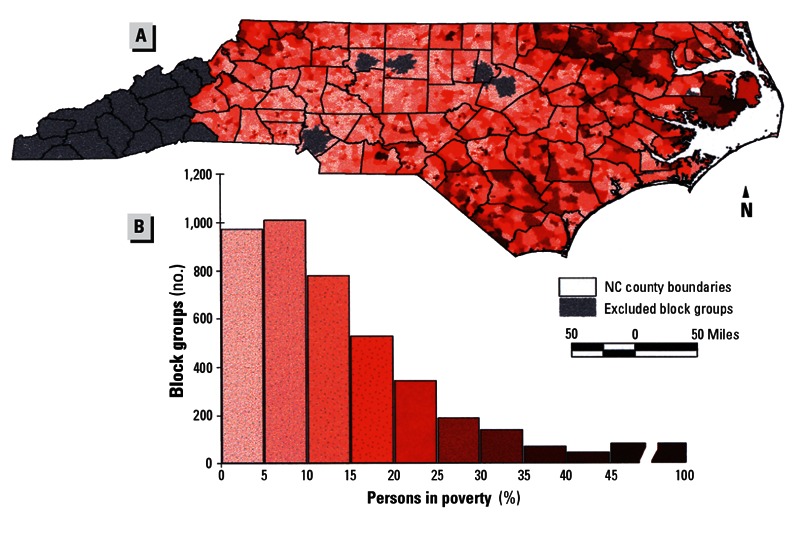
Maps from an older study show distributions of poverty, minority residents, and hog CAFOs in North Carolina as of 1998–2000. Little has changed appreciably since then. Source: Wing et al. (2000)^20^

**Figure:**
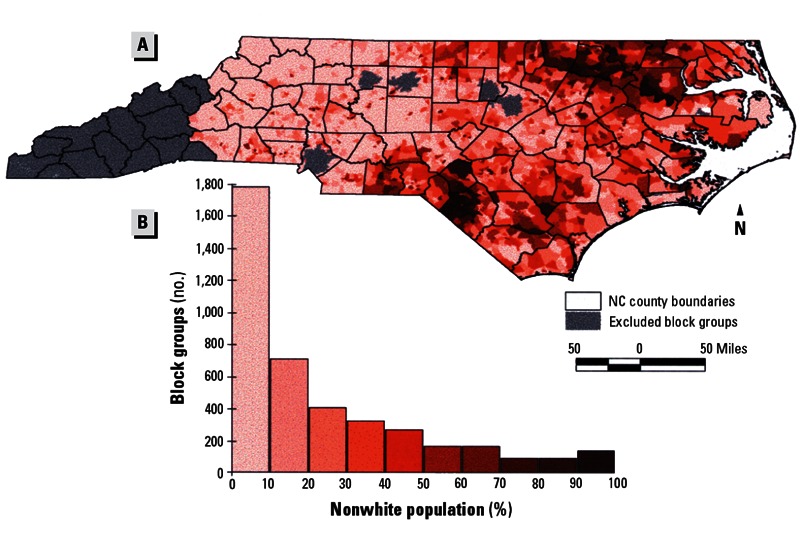
Maps from an older study show distributions of poverty, minority residents, and hog CAFOs in North Carolina as of 1998–2000. Little has changed appreciably since then. Source: Wing et al. (2000)^20^

The industry’s rapid growth in the state followed the passage of these bills, causing a major shift in the state’s hog farming. In 1982 every county in North Carolina but one had a commercial hog farm; by 1997, 95% of hog farms were located in the eastern counties of the coastal plain.^32^

Today the North Carolina hog herd, all told, numbers around 9–10 million animals annually, according to the state Department of Agriculture and Consumer Services.^41^ This results in an enormous amount of manure, with each hog producing an estimated four to eight times as much feces as a human.^32^^,^^42^ In 2008 the Government Accountability Office reported that some 7.5 million hogs in five eastern North Carolina counties produced an estimated 15.5 million tons of waste per year, and that in one year a single 80,000-head facility could create 1.5 times the waste of the city of Philadelphia.^43^

The lagoons in which this waste is stored contain pathogens such as *Salmonella*, insecticides, antimicrobial agents and other pharmaceuticals, and nutrients that cause widespread pollution and impairment of watersheds across the coastal plain.^44^^,^^45^^,^^46^ Much concern has been raised over antibiotic-resistant bacteria that result from CAFO animals’ near-continual exposure to subtherapeutic doses of antibiotics as an inexpensive means to prevent disease and promote growth.^47^^,^^48^

Whereas human sewage is treated with chemical and mechanical filtration before being released into the environment, CAFOs channel waste from hog houses into pits or lagoons, where it is stored untreated until it is applied to land. All lagoons leach to some degree,^49^^,^^50^^,^^51^ and during hurricanes and storms they can overflow or burst, spilling raw sewage onto the landscape and into waterways. In 1995 an eight-acre lagoon ruptured, spilling 22 million gallons of manure into North Carolina’s New River, killing millions of fish and other organisms; other spills followed that summer.^52^^,^^53^ Even without spills, ammonia and nitrates may seep into groundwater, especially in the coastal plain where the water table is near the surface.^32^^,^^54^

## Odors, Plumes, and Toxics

Although more research is needed on the impact of CAFO emissions on susceptible groups of people,^10^ studies have linked hog odors and air pollution from the associated odor plume with adverse effects on health and quality of life.^55^ Wilson, who has documented environmental justice issues surrounding hog farms in North Carolina and Mississippi, explains that CAFO emissions go beyond bad smells. “It’s much more complex than that,” he says. “You have exposures through air, water, and soil. You have … inhalation, ingestion, and dermal exposures. People have been exposed to multiple chemicals: hydrogen sulfide, particulate matter, endotoxins, nitrogenous compounds. Then you have a plume that moves; what gets into the air gets into the water. You have runoff from spray fields. These are complex exposure profiles.”

**Figure:**
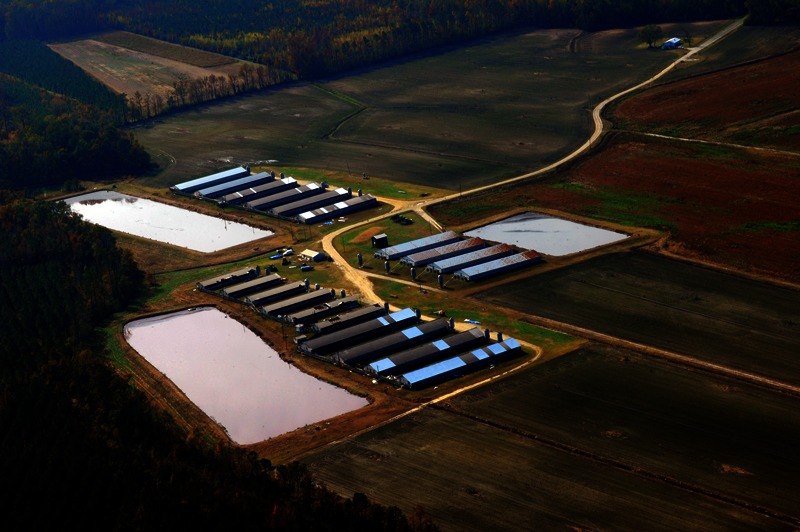
CAFOs apply accumulated animal waste to spray fields of Bermuda grass or field crops located around the barns and lagoons. © 2013 Donn Young Photography

**Figure:**
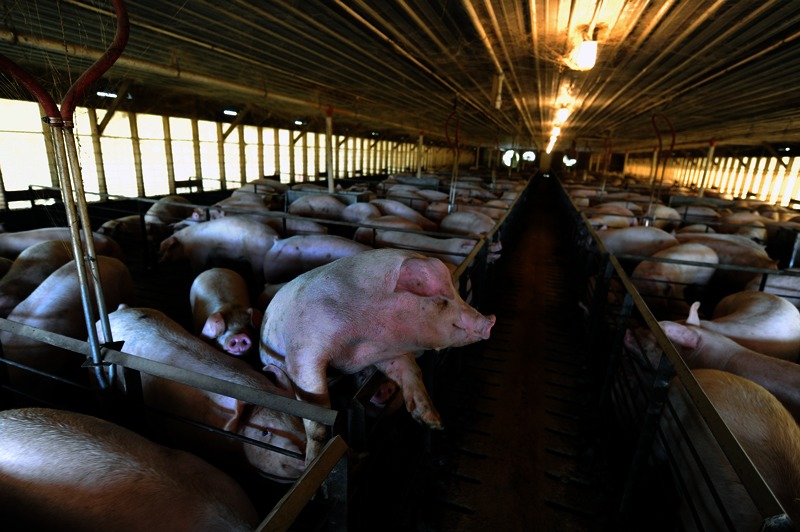
Hogs are packed tightly inside CAFOs like this one in Princeton, North Carolina. © 2013 Donn Young Photography

University of North Carolina epidemiology professor Steve Wing and colleagues have reported that waste odor frequently prevents local residents from spending time outdoors, opening windows, putting laundry out to dry, or inviting visitors over.^9^^,^^56^ In the Community Health Effects of Industrial Hog Operations study, a repeated-measures, community-driven project, Wing and colleagues enrolled 102 individuals in 16 communities to sit outside twice a day, recording odor strength and symptoms such as eye irritation and difficulty breathing. Participants self-monitored aspects of their physical health, including blood pressure and lung function, and also used mobile air pollution monitors to collect data on levels of hydrogen sulfide, endotoxin, coarse particulate matter (PM_10_), and semivolatile compounds in particle phase within each neighborhood.

The researchers found that hydrogen sulfide levels were strongly related to odor.^57^ Furthermore, measures of odor, endotoxin, hydrogen sulfide, and PM_10_ were associated, variously, with increased respiratory difficulty, sore throat, chest tightness, nausea, and eye irritation,^58^ whereas hydrogen sulfide and semivolatile particles were linked to reports of feeling stressed, annoyed, nervous, and anxious.^13^

Most recently, Wing reported associations between blood pressure increases and increased odor and hydrogen sulfide.^14^ “In this primarily African-American population, in a region that is known historically as the Stroke Belt because of very high rates of death from cerebrovascular disease, we don’t need environmental exposures that are leading to additional blood pressure increases,” Wing says.

**Figure:**
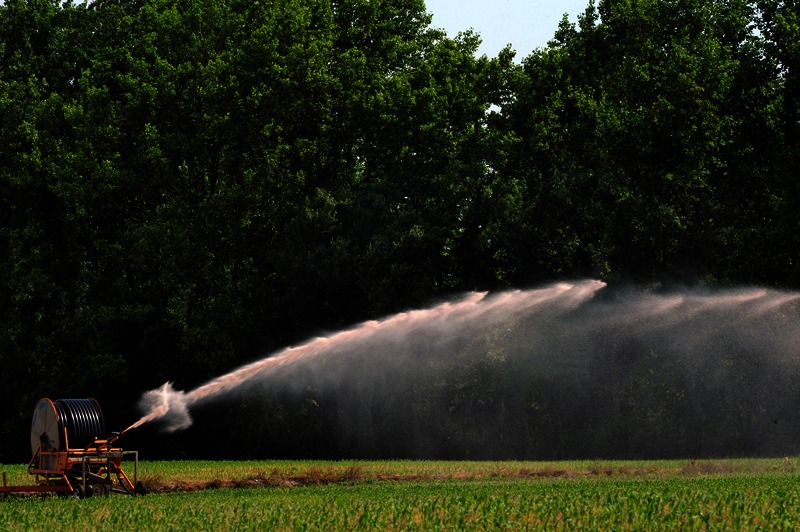
Hog waste being applied to sprayfields near Warsaw, North Carolina. Nutrients, pathogens, heavy metals, and other potentially toxic agents in the waste can make their way into local watersheds, with implications for drinking water and aquatic ecosystems. © 2013 Donn Young Photography

Because these communities are typically impoverished and lack political clout, they often have little means to fight back.^59^ “It creates a major burden on communities when they have few legal protections,” says Wilson. However, getting communities involved in data collection has empowered citizens.^59^ “When we train residents to do sampling, they understand the science of the process,” says Wilson. “They can go to the town council, they can go to the media, they can explain it. That’s powerful. It helps build up a community’s ability to be more involved in decision making.”

## Who Looks After Residents’ Health?

The shift to CAFOs happened so fast that regulations and laws protecting human health and the environment have not caught up with the changing face of animal husbandry. A 2013 report revealed that despite the highly localized health impacts associated with CAFOs, local and state health departments generally do not have jurisdiction over them; instead, that responsibility is typically held by state environmental or natural resource agencies.^60^ Jillian Fry, a researcher at the Johns Hopkins Center for a Livable Future who was lead author on that report, says, “The agencies responsible for regulating CAFOs—their mission is not to protect human health.”

Fry says the study was inspired by a CAFO expansion meeting she attended with a colleague. A proponent of the expansion stood up at the meeting and stated that if hog farms caused health concerns, the health department would make the community aware; therefore, there was nothing to worry about. “I knew … that the health department was not involved, so we wanted to see what the situation was in other parts of the country,” Fry says.

She and her colleagues interviewed health department staff in eight states and found that most health departments did not deal with CAFO issues. Either they lacked the jurisdiction, had no budget or expertise, or were dealing with political pressure. Fry says, “Even if a health department thinks this is a really important issue, we’re hearing from a lot of them, ‘We’re aware of the science, we know of the problem, but it’s the political barriers.’”

**Figure:**
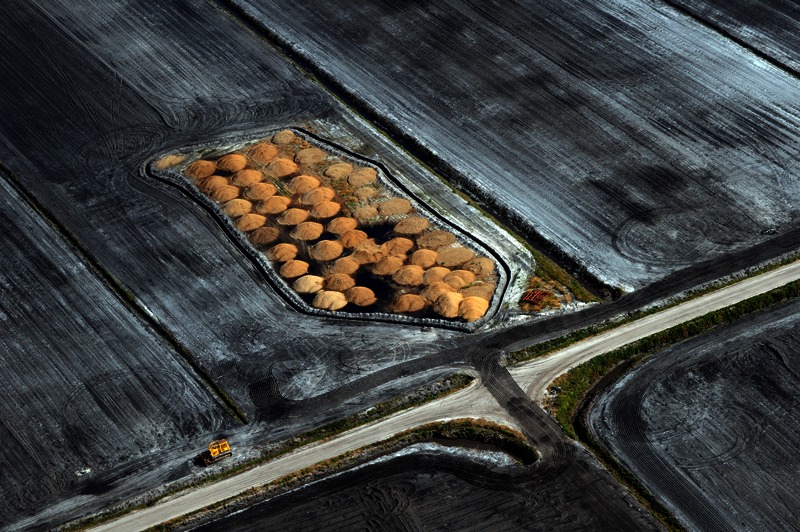
Piles of what is believed to be poultry litter in a field near New Bern, North Carolina. A state moratorium on new hog CAFOs has resulted in the construction of more poultry CAFOs, according to researcher Steve Wing. © 2013 Donn Young Photography

The survey also found that community members did not get very far with inquiries. “We asked community members, ‘Was there ever a time you contacted a health department and they addressed your complaint?’ They all said no,” says Fry. “They were almost always referred to another agency, or maybe they would look into it and hit a barrier.”

## An Eye to the Future

With accumulating scientific evidence over the environmental and community health impacts of hog CAFOs and extensive media coverage of ruptured lagoons, opposition crescendoed in the mid-1990s. In 1997 North Carolina passed a law prohibiting the expansion of existing hog operations and placing a temporary moratorium on new hog CAFOs,^61^ although permits in the hopper were approved. The moratorium became permanent in 2007 with the Swine Farm Environmental Performance Standards Act, which banned new lagoons and mandated that any new or expanded CAFOs must use environmentally superior technologies (ESTs) to substantially reduce emissions and prevent waste discharges into surface and ground waters.^62^ The 2007 law provided for a substantial cost-share for operators to upgrade their lagoons and implement ESTs, yet only 11 of 2,200 have applied, and only 8 have participated.^63^^,^^64^

Although the act limited growth of new hog facilities, it didn’t clean up existing ones, says Wing. Local residents still deal daily with odor and pollutants in the vicinity of hog farms. The moratorium also catalyzed other changes whose impact is yet to be fully realized. “More poultry facilities have been built,” Wing says. “That brings up other issues such as the spread of microbes between species.”

Another milestone occurred when Smithfield Foods, Inc., entered into an agreement with the state Attorney General in 2000 after dozens of lagoons ruptured during Hurricane Floyd, resulting in Clean Water Act violations.^65^ Smithfield Foods agreed to pay $15 million toward research on ESTs and $50 million toward environmental enhancement.^66^^,^^67^ Premium Standard Farms, a subsidiary of Smithfield Foods, later voluntarily added $2.1 million toward the agreement for EST research and development.^68^ If an EST were found to be both economically feasible and environmentally superior in five categories, the companies agreed to implement it at each of the farms they owned, although not at farms they subcontracted. (Mike Williams, director of the Animal and Poultry Waste Management Center at North Carolina State University and supervisor of the agreement, says an estimated 5–10% of North Carolina hog farms are company-owned.)

After phase 1 of development, only one of the new technologies examined—the Super Soil System (since renamed Terra Blue)—met all five environmental standards, but it was deemed uneconomical. Improvements made during phase 2 reduced the cost but not enough to meet the economic criteria. The project is now in the final weeks of phase 3. “If the process shows that it does meet bona fide EST status and economic criteria, then the agreement states [farms have a certain] amount of time to implement,” Williams says.

In 2011 the state passed a bill that allows hog CAFOs to upgrade their buildings without needing to upgrade their waste management systems or use ESTs, counter to the previous decade’s mandates.^69^ Some critics have called this a loophole, given that the 2007 law stipulated hog farmers were supposed to implement ESTs if they wanted to increase herd size or install new buildings.^70^

At the same time, the handful of pioneers who are implementing ESTs are creating what could be the future of hog farming.^71^ In one of those projects, Google has partnered with Duke University and Duke Energy to turn Yadkin County’s Loyd Ray Farms into a sustainable operation that generates renewable energy and carbon offsets.^72^ The 8,600-head finishing farm captures methane from its hog waste using an anaerobic digester. The methane provides fuel to run a microturbine that powers part of the farm and supports components that reduce odors, nutrients, pathogens, and heavy metals. Google and Duke University share the carbon credits, while Duke Energy receives renewable energy certificates (credits for generating renewable energy that are sold separately from the actual electricity produced^73^). Although projects like these so far make up only a tiny fraction of the market, their experimental approach could lead the way toward hog farms becoming better neighbors.
