# Erratum: “CAFOs and Environmental Justice: The Case of North Carolina”

**DOI:** 10.1289/ehp.121-a210

**Published:** 2013-07-01

The June 2013 News article “CAFOs and Environmental Justice: The Case of North Carolina” [Environ Health Perspect 121:A182–A189 (2013)] referred to farms that companies “co-own with farmers.” These farms should have been referred to as “company-owned.” *EHP* regrets the error.

